# Endometrial Thickness Is a Risk Factor for Singleton Low Birth Weight From Single Blastocyst Transfer: A Retrospective Cohort Study

**DOI:** 10.3389/fendo.2021.730512

**Published:** 2021-09-27

**Authors:** Mingze Du, Junwei Zhang, Manman Liu, Yichun Guan, Xingling Wang

**Affiliations:** The Reproductive Center, The Third Affiliated Hospital of Zhengzhou University, Zhengzhou, China

**Keywords:** low birth weight, endometrial thickness, blastocyst, birthweight, *in vitro* fertilization

## Abstract

**Objective:**

To explore whether endometrial thickness (EMT) ≤7.5 mm is associated with singleton low birth weight (LBW) from single fresh blastocyst transfer.

**Methods:**

This was a retrospective cohort study. Only women ≤ 40 years old who underwent single fresh blastocyst transfer and had singleton live births were included in the study. LBW was the primary outcome of this study. Neonatal malformation was the secondary outcome. Logistic regression was used to evaluate the association between (EMT) ≤7.5 mm and singleton LBW.

**Results:**

A total number of 2847 women met the study inclusion criteria. The neonatal birthweight in the EMT ≤7.5 mm group was significantly lower than that in the EMT 7.6~12.0 mm and EMT >12.0 mm group (P<0.001). The rate of LBW in the EMT ≤7.5 mm group was 24.9%, which was significantly higher than the 4.0% in the EMT 7.6~12.0 mm group and the 5.3% in the EMT >12.0 mm group (P<0.001). The total neonatal malformation rate was similar between the groups (1.1%, 0.8% and 1.5%, P=0.21). After logistic regression analysis, EMT ≤7.5 mm was found to be an independent risk factor for LBW (adjusted odds ratio [AOR]: 4.39, 95% CI: 1.85_˜_10.46, P<0.001).

**Conclusion:**

EMT ≤7.5 mm on the hCG trigger day is an independent risk factor for LBW in singleton pregnancies from single fresh blastocyst transfer. The neonatal birthweight in the EMT ≤7.5 mm group was significantly lower than that in the EMT 7.6~12.0 mm and EMT >12.0 mm groups. The total neonatal malformation rate was comparable between the groups.

## Introduction

Infertility affects approximately 10% of reproductive-aged couples worldwide (1). *In vitro* fertilization (IVF) is widely used and has become the most effective treatment for infertility caused by tubal or other factors as well as unexplained infertility (2). However, adverse pregnancy and perinatal outcomes, such as low birth weight (LBW), preterm delivery, pregnancy-induced hypertension and gestational diabetes, are increased, even for singleton births in IVF pregnancies (3–5). The exact biological mechanism leading to adverse perinatal outcomes is unclear. Some studies have shown that infertility itself is the main reason for the poor perinatal outcomes of assisted reproductive technology (ART) singleton offspring (6). However, an increasing number of studies have shown that the process of ART, including exposure to superphysiological doses of hormones and embryo manipulation in the laboratory, may have an adverse effect on the perinatal outcome (7–10). Furthermore, defective placentation, particularly in patients with a thin endometrium, may be an important cause of these poor perinatal outcomes (11, 12). There is no exact definition of “thin endometrium”, and most studies have reported cutoff values of 7 mm, 7.5 mm or 8 mm (13–17). EMT is an independent factor affecting the ART pregnancy rate (18). In recent years, studies have shown that EMT is not only related to the pregnancy rate but also to perinatal complications and offspring safety (13–15). However, there are few related studies and limited data, and cleavage-stage embryos and blastocysts have not been studied and analyzed separately. Different stages of embryo development may have different effects on offspring safety (19, 20). Therefore, in this study, we selected patients with single fresh blastocyst transfer and analyzed the effect of EMT on the LBW of singleton offspring.

## Materials and Methods

### Study Design and Population

This is a retrospective study that was approved by the review board of The Third Affiliated Hospital of Zhengzhou University. All patients who initiated their first *in vitro* fertilization (IVF)/intracytoplasmic sperm injection (ICSI) cycles at the Reproductive Center of Third Affiliated Hospital of Zhengzhou University between January 2016 and February 2020 were analyzed for potential inclusion. Inclusion criteria were women < 40 years old, single fresh blastocyst transfer (D5/D6) and singleton live birth. Cycles with oocyte donation, vanishing twins, adenomyosis, uterine malformations, endometrial polyps, preimplantation genetic testing (PGT) or incomplete records were excluded.

### Clinical and Laboratory Protocols

All the women underwent either GnRH agonist (GnRH-a) or flexible GnRH antagonist (GnRH-anti) protocols. The details of controlled ovarian hyperstimulation (COH) were described in our previous study (3). For the GnRH-a protocol, protracted downregulation with GnRH-a (Diphereline, lpsen, France) 3.75 mg was performed on the second or third day of the menstrual cycle, followed by daily use of recombinant follicle-stimulating hormone (rFSH), which was based on the ovarian response. In the flexible GnRH-anti group, rFSH was initiated on the second or third day of the menstrual cycle. Injection of GnRH-anti at 0.25 mg/day commenced once the diameter of the dominant follicle reached 14 mm and was continued up to the trigger day.

For both protocols, the dose of rFSH was adjusted according to the follicle response. As soon as the diameter of the dominant follicle was greater than 20 mm or when at least three follicles reached 18 mm, ovulation induction was triggered with 5000 to 10000 IU hCG (Lizhu Pharmaceutical Trading, China). Oocyte retrieval was performed 36 hours later. Based on the sperm quality, conventional IVF or ICSI was performed approximately 4 to 6 hours after follicular aspiration. In our study, all women underwent single fresh blastocyst transfer on the fifth day after fertilization. Routine corpus luteum support was initially provided on the day of oocyte retrieval, mainly, oral dydrogesterone (10 mg twice daily) (Abbott Co. America), and intravaginal administration of 90 mg of a progesterone sustained-release vaginal gel (Merck Co. Germany) was given. Corpus luteum support was performed at least until 55 days after transplantation if pregnancy occurred.

### EMT Assessment and Grouping

EMT was assessed by transvaginal ultrasound performed by three doctors in our reproductive center with extensive experience. EMT was measured in the sagittal plane on the hCG trigger day. The distance between the hyperechogenic interfaces between the endometrium and the myometrium was recorded approximately 1 centimeter beneath the uterine fundus. The EMT was recorded and analyzed in millimeters. Patients were categorized into three groups depending on their EMTs: ≤7.5 mm, 7.6~12.0 mm and >12.0 mm. These cutoffs were selected as per those in previous studies (13–15).

### Outcome Measures and Definition

LBW was defined as a neonatal birth weight less than 2500 g and was the primary outcome of this study (21). The secondary outcome measures were neonatal birthweight and neonatal congenital malformations, including trisomy 13/18/21, congenital heart disease, polydactyly/syndactyly and other disorders.

### Statistical Analysis

All of the data analysis in our study were obtained *via* review of our reproductive center’s medical records.

The one-sample Kolmogorov–Smirnov test was used to check for normality of continuous variables. The Wilcoxon rank-sum test was used to assess between-group differences in continuous variables with abnormal distributions, and these variables are expressed as the median (P25, P75). Categorical variables are presented as the number of cases (n) and the percentage (%). The means obtained from chi-square analyses were used to assess the differences between groups with Fisher’s exact test when necessary. For LBW, logistic regression was used to adjust for the baseline characteristics between groups. Adjusted odds ratios (AORs) with 95% confidence intervals (CIs) were calculated. All statistical management and analyses were performed using SPSS software, version 24.0. Statistical significance was set at p<0.05.

## Results

### Study Population

A total number of 2847 women who underwent single fresh blastocyst transfer and had singleton live births met the study inclusion criteria. 181 women had EMT ≤7.5 mm, 1714 women had EMT 7.6~12.0 mm and 952 women had EMT >12.0 mm.

### Characteristics of the Study Groups

**Table 1** shows the demographic and clinical characteristics among the three groups. There were no statistically significant differences in between the groups for maternal age, body mass index (BMI), duration of infertility, type of infertility, infertility diagnosis, basal serum FSH level, number of antral follicle counts, fertilization method, COH protocols, duration of ovarian stimulation, dosage of gonadotropins, serum estradiol on the trigger day or number of oocytes retrieved.

**Table 1:** Comparison of demographic and clinical characteristics among the three groups.

Item	EMT ≤ 7.5 mm	EMT 7.6-12 mm	EMT 12> mm	P value
No. of cases	181	1714	952	
Maternal age (year)	30 (28,35)	29 (27,33)	29 (27,33)	0.12
Body mass index(kg/m^2^)	23.4 (20.8,25.0)	22.7 (20.7,25.0)	22.8 (20.9,25.3)	0.46
Duration of infertility (year)	3.0 (1.5,4.0)	3.0 (2.0,5.0)	3.0 (2.0,5.0)	0.29
Type of infertility(%)				0.25
Primary infertility	50.3 (91/181)	48.4 (829/1714)	45.4 (432/952)	
Secondary infertility	49.7 (90/181)	51.6 (885/1714)	54.6 (520/952)	
History of miscarriage(%)	44.4 (40/90)	44.0 (389/885)	46.2 (240/520)	0.73
History of ectopic pregnancy(%)	13.3 (12/90)	10.2 (90/885)	11.0 (57/520)	0.62
Infertility diagnosis(%)				0.06
Tubal factor	38.1 (69/181)	40.7 (698/1714)	35.0 (333/952)	
Male factor	26.5 (48/181)	24.0 (411/1714)	27.0 (257/952)	
Others	35.4 (64/181)	35.3 (605/1714)	38.0 (362/952)	
Basal FSH(IU/L)	6.6 (5.2,7.8)	6.7 (5.5,8.1)	6.8 (5.6,8.0)	0.33
Number of antral follicle count	15 (10,18)	15 (11,19)	15 (11,19)	0.47
Methods of ART (%)				0.07
IVF	73.5 (133/181)	73.0 (1251/1714)	68.9 (656/952)	
ICSI	26.5 (48/181)	27.0 (463/1714)	31.1 (296/952)	
COH protocols (%)				0.88
GnRH-a protocol	70.7 (128/181)	71.8 (1230/1714)	70.9 (675/952)	
GnRH-anti protocol	29.3 (53/181)	28.2 (484/1714)	29.1 (277/952)	
Duration of ovarian stimulation (d)	12 (10,14)	13 (12,15)	13 (12,15)	0.17
Dosage of gonadotropins(IU)	2550 (2025,3562)	2475 (1950,3300)	2475 (1900,3300)	0.87
Serum estradiol on the trigger day (pg/ml)	3003.0 (1966.5,4532.5)	3077.9 (2097.5,4607.3)	3190.5 (3123.5,4677.3)	0.39
No. of oocytes retrieved	10 (7,13)	11 (8,13)	11 (8,14)	0.10

Data are presented as median (P25, P75) for continuous variable and n (%) for categorical variable.

### Neonatal Outcomes

The neonatal birthweight in the EMT ≤7.5 mm group was significantly lower than that in the EMT 7.6~12.0 mm and EMT >12.0 mm group (EMT ≤7.5 mm, 3000 (2525,3350); EMT 7.6~12.0 mm, 3350 (3050,3650); EMT >12.0 mm, 3400 (3100,3690), P<0.001). The gender of the newborns was comparable between groups (P=0.06). The rate of LBW in the EMT ≤7.5 mm group was 24.9%, which was significantly higher than the 4.0% in the EMT 7.6~12.0 mm and 5.3% in the EMT >12.0 mm group (P<0.001). The cesarean section ratio (60.8%, 65.0% and 68%, P=0.11) and total neonatal malformation rate was similar between the groups (1.1%, 0.8% and 1.5%, P=0.21) (**Table 2**).

**Table 2:** Comparison of neonatal birthweight, gender and malformations among the three groups.

	EMT ≤ 7.5 mm	EMT 7.6-12 mm	EMT 12> mm	P value
No. of cases	181	1714	952	
Birthweight (g)	3000 (2525,3350)*	3350 (3050,3650)	3400 (3100,3690)	<0.001
Cesarean section ratio(%)	60.8 (110/181)	65.0 (1114/1714)	68.0 (647/952)	0.11
Gender of newborn (%)				0.06
Male	55.2 (100/181)	52.2 (894/1714)	56.9 (542/952)	
Female	44.8 (81/181)	47.8 (820/1714)	43.1 (410/952)	
LBW (%)	24.9 (45/181)*	4.0 (69/1714)	5.3 (50/952)	<0.001
Neonatal malformations (%)	1.1 (2/181)	0.8 (13/1714)	1.5 (14/952)	0.21

Data are presented as median(P25, P75) for continuous variable and n (%) for categorical variable; * shows that there is a statistically significant difference between EMT ≤ 7.5 mm and EMT 7.6-12 mm, EMT ≤ 7.5 mm and EMT 12> mm.

A logistic regression analysis was conducted to adjust for the influence of the confounding factors on the main outcome-LBW. The regression model included the following factors: maternal age (continuous variable), BMI (continuous variable), type of infertility (primary/secondary infertility), infertility diagnosis (tubal/male/others), COH protocols (GnRH-a protocol/GnRH-anti protocol), serum estradiol on the trigger day (<4000 pg/ml≥4000 pg/ml), EMT (≤7.5/7.6~12.0/>12.0 mm), number of gestational weeks (continuous variable) and sex of the newborn (male/female). Compared with EMT 7.6~12.0 mm, EMT ≤ 7.5 mm was an independent risk factor for LBW (AOR: 4.39, 95% CI: 1.85˜10.46, P<0.001). In addition, BMI (AOR: 0.91, 95% CI: 0.84˜0.99, P=0.03), serum estradiol on the trigger day (AOR: 1.87, 95% CI: 1.16˜2.21, P=0.03), gestational weeks (AOR: 0.37, 95% CI: 0.32˜0.42, P<0.001) and the gender of the newborn (AOR: 1.99, 95% CI: 1.19˜3.33, P=0.01) were risk factors for LBW. The detailed data are described in **Table 3**.

**Table 3:** Logistic regression analysis to account for confounding variables of low birth weight.

	AOR (95% CI)	P value
Age	0.97 (0.90˜1.04)	0.31
Body mass index	0.91 (0.84˜0.99)	0.03
Type of infertility (primary/secondary)	1.66 (0.93˜2.93)	0.09
Infertility diagnosis (tubal/male/others)	1.11 (0.82˜1.50)	0.52
Method of ART (IVF/ICSI)	1.06 (0.59˜1.92)	0.74
COH protocols (GnRH-a protocol/GnRH-anti protocol)	1.21 (0.90˜1.62)	0.22
The serum estradiol on the trigger day (<4000 pg/ml≥4000 pg/ml)	1.87 (1.16˜2.21)	0.03
EMT (mm)		<0.001
7.6~12.0	1	
>12.0	0.90 (0.51˜1.59)	0.72
≤7.5	4.39 (1.85˜10.46)	<0.001
Gestational weeks	0.37 (0.32˜0.42)	<0.001
Gender of newborn (male/female)	1.99 (1.19˜3.33)	0.01

Variable entered in the logistics regression model listed. AOR, adjusted odds ratio; CI, confidence interval. EMT, endometrial thickness.

## Discussion

### Main Findings

In this single-center retrospective cohort study, EMT (≤7.5 mm) on the hCG trigger day was an independent risk factor for LBW of a singleton live birth from a single fresh blastocyst transfer. The neonatal birthweight in the EMT ≤7.5 mm group was significantly lower than that in the EMT 7.6~12.0 mm and EMT >12.0 mm groups. The total neonatal malformation rate was comparable between groups.

### Comparison With Other Studies

The first study to explore the relationship between EMT and perinatal outcome was performed by Chung et al. (22). This study found that suboptimal endometrial development is associated with adverse outcomes in pregnancies achieved through IVF (22). Another early study explored the relationship between EMT and the risk of placenta previa. Rombauts et al. found that the risk of placenta previa was significantly higher in women with EMT>12 mm than in women with an endometrial thickness of <9 mm (23). A large retrospective cohort study including 6181 singleton newborns found that a thin endometrium was associated with a lower mean birthweight and birthweight Z-score (15). Recently, Guo et al. (13) reported that the risk of being small for gestational age (SGA) was increased approximately twofold in women with EMT ≤ 7.5 mm compared with women with EMT >12 mm after fresh embryo transfer. However, to the best of our knowledge, the current study did not analyze the effect of EMT on the hCG trigger day on the neonatal birthweight from single fresh blastocyst transfer. We know that the different stages of embryo transfer, the cleavage or blastocyst stage, may have different effects on perinatal outcomes (19, 24, 25).

However, single blastocyst transfer, compared with cleavage-stage embryo transfer, can increase the pregnancy rate, and has a lower risk of multiple births (26). Moreover, single blastocyst transfer is increasingly widely used in clinical practice and may become the recommended transplantation strategy. Therefore, to reduce the influence of confounding factors, it is necessary to further analyze the impact of EMT of single blastocyst transfer on the safety of the offspring. In the present study, EMT (≤7.5 mm) was an independent risk factor for LBW of a singleton live birth from a single fresh blastocyst transfer.

### Strengths and Limitations

In the current retrospective study, to minimize the influence of confounding factors, only single fresh blastocyst transfer was included. After analyzing and adjusting for confounding factors by EMT grouping, we found that EMT ≤ 7.5 mm is an independent risk factor for LBW and further confirmed the influence of EMT on neonatal birthweight. It is an important supplement to current clinical research. To the best of our knowledge, this is the first study to explore the effect of EMT on singleton LBW from single fresh blastocyst transfer. Only single-center data were included to minimize the influence of confounding factors caused by different EMT measurements, clinical protocols, and laboratory operations. However, this investigation also has certain limitations. It was a retrospective cohort study and did not further explore the relevant biological mechanism by which EMT affects the incidence of LBW.

### Interpretation

The specific biological mechanism of the impact of EMT on newborn weight and perinatal outcomes is still unclear and likely complex. Research on the main mechanism focuses on the oxygen concentration. After ovulation, the uterine spiral arteries contract, and the blood flow on the surface of the endometrium decreases, thereby reducing the oxygen concentration of the functional epithelium during embryo implantation (27). In early pregnancy, hypoxic tension is one of the main conditions for normal embryo implantation and fetal development (28). However, the thinning or lack of a functional layer may cause more blood vessel growth and higher oxygen concentrations in the basal endometrium of the embryo. The subsequent high oxygen tension may affect embryo implantation and placental development, thereby affecting the growth and development of the fetus (29, 30). Another mechanism is related to spiral arterial revascularization. In early pregnancy, spiral arterial vascular remodeling is an important factor in determining placental blood perfusion. The lack of vascular remodeling will affect the blood supply of the placenta and eventually lead to perinatal complications, such as fetal growth restriction and pregnancy-related hypertension (31, 32). The uterine artery blood flow resistance of a thin endometrium is high, and there is vascular dysplasia (12). These changes may affect spiral arteries vascular remodeling, thereby affecting the development of the placenta and causing poor perinatal outcomes (12, 15).

## Conclusion

In conclusion, this study showed that EMT (≤7.5 mm) was significantly correlated with neonatal birthweight and was an independent risk factor for singleton LBW from fresh single blastocyst transfer. In addition, the neonatal birth weight in the EMT ≤7.5 mm group was significantly lower than that in the EMT 7.6~12.0 mm and EMT >12.0 mm groups. This finding may be related to spiral arterial vascular remodeling and placental development, and further research is needed to explore its related biological mechanism. Therefore, for patients with EMT ≤ 7.5 mm, the perinatal period may require more attention from obstetricians and pediatricians.

## Data Availability Statement

The raw data supporting the conclusions of this article will be made available by the authors, without undue reservation.

## Ethics Statement

This study was approved by the ethics committee of The Third Affiliated Hospital of Zhengzhou University. Written informed consent for participation was not required for this study in accordance with the national legislation and the institutional requirements.

## Author Contributions

XW designed the study. MD and JZ were involved in the data extraction and analysis. ML reviewed the data. MD and JZ was involved in drafting this article. MD and YG revised this article. All authors contributed to the article and approved the submitted version.

## Conflict of Interest

The authors declare that the research was conducted in the absence of any commercial or financial relationships that could be construed as a potential conflict of interest.

## Publisher’s Note

All claims expressed in this article are solely those of the authors and do not necessarily represent those of their affiliated organizations, or those of the publisher, the editors and the reviewers. Any product that may be evaluated in this article, or claim that may be made by its manufacturer, is not guaranteed or endorsed by the publisher.
